# Crop detection technologies, mechanical weeding executive parts and working performance of intelligent mechanical weeding: a review

**DOI:** 10.3389/fpls.2024.1361002

**Published:** 2024-03-14

**Authors:** Meiqi Xiang, Minghao Qu, Gang Wang, Zhongyang Ma, Xuegeng Chen, Zihao Zhou, Jiangtao Qi, Xiaomei Gao, Hailan Li, Honglei Jia

**Affiliations:** College of Biological and Agricultural Engineering, Jilin University, Changchun, China

**Keywords:** physical weed control, identification and positioning methods, applicable scenario, applicable limitations, future trends

## Abstract

Weeding is a key link in agricultural production. Intelligent mechanical weeding is recognized as environmentally friendly, and it profoundly alleviates labor intensity compared with manual hand weeding. While intelligent mechanical weeding can be implemented only when a large number of disciplines are intersected and integrated. This article reviewed two important aspects of intelligent mechanical weeding. The first one was detection technology for crops and weeds. The contact sensors, non-contact sensors and machine vision play pivotal roles in supporting crop detection, which are used for guiding the movements of mechanical weeding executive parts. The second one was mechanical weeding executive part, which include hoes, spring teeth, fingers, brushes, swing and rotational executive parts, these parts were created to adapt to different soil conditions and crop agronomy. It is a fact that intelligent mechanical weeding is not widely applied yet, this review also analyzed the related reasons. We found that compared with the biochemical sprayer, intelligent mechanical weeding has two inevitable limitations: The higher technology cost and lower working efficiency. And some conclusions were commented objectively in the end.

## 1 Introduction

There is a long history of weeds negatively affect agricultural production, weeds compete against crops for nutrients, sunlight, and water, which lowers crop production and quality (Nichols et al., 2015). According to the publicly available data, prior to the widespread application of herbicides, the average yearly loss of production of Chinese crops, including grain, cotton, oil, and other crops, caused by field weeds was 3,419,609 tons, 61,065 tons, 189,304 tons, and 515,256 tons, respectively (Ministry of Agriculture and Rural Affairs of the PRC, 2024). Gharde et al. (2018) also calculated an average number that the yield losses caused by weeds of four major field crops: 15 to 66% in direct-seeded rice, 18 to 65% in maize, 50 to 76% in soybean, and 45 to 71% in groundnut. The annual weeding cost as running into the $100 billion us dollars globally (Kraehmer and Baur, 2013). Weeding is essential for both economic development and agricultural productivity.

Manual weeding, biological weeding and chemical weeding have been applied in agricultural production for a long time (Li et al., 2022b). However, traditional manual weeding is labor-intensive, which is not in line with the development of modern agriculture. According to a study conducted in Zambia, the average labor requirement is 50.25 person-days per hectare (Lee and Thierfelder, 2017). In the Netherlands, the mean input of manual weeding in organic row crops is ca. 45 h·ha^-1^ for planted vegetables, but increases to more than 175 h·ha^-1^ for direct-sown onion (*Allium cepa* L.) under field conditions. Compared with manual weeding, biochemical weeding has higher efficiency and better weeding performance, which gradually becomes the most widely used weeding method worldwide. Nonetheless, the constant-rate and indiscriminate application of chemical weeding easily lead to weed resistance, cause environmental contamination and public health problems (Laursen et al., 2016; Wu et al., 2020; Villette et al., 2022). In the 20th century, due to intensification and automation, agricultural mechanization has increased significantly over the years, and mechanical weeding since no biochemical spray is applied, there will be no herbicide residue, as a green method that protects the environment has begun to be widely concerned (Bechar and Vigneault, 2016). Currently, with the advent of desire for organic agriculture and precision agriculture in tandem, mechanical weeding poses a new challenge to intelligent development (Zhang et al., 2021). Intelligent mechanical weeding can perfectly supplement the flaws of manual weeding and biochemical weeding, which can profoundly improve the crop quality.

Generally speaking, the intelligent mechanical weeding machine is a robot, which can realize weeding like human beings, they can distinguish weeds and crops, and locate their positions (Jin et al., 2022). Intelligent mechanical weeding is an organic combination of computer science, electronics, machinery and many other disciplines. Seedling and weed detection are pre-technologies for intelligent weeding, and then all kinds of WEP could play their important roles. Mechanical weeding includes inter-row weeding and intra-row weeding. Among them, the inter-row weeding is easy to deal with because of the absence of interference from the crop row; nevertheless, the intra-row weeding requires real-time crops avoidance to prevent damage. Therefore, how to achieve intra-row weeding is a challenging research in mechanical weeding (Quan et al., 2021). Usually, there are two major methods for intelligent mechanical weeding. In one situation, the intelligent weeding machine could detect the weeds and then remove them directly. Another scenario is that the intelligent weeding machine detects the crop, then avoids the crop and executes the weeding movements in locations without crop seedlings. Since crops are almost mechanically planted in rows nowadays, their positions are more uniform then weeds, so the latter scenario is more cost-effective in real applications. Such as the designs of Quan et al. (2022); Garford (2023); Poulsen (2023b) and the German company K.U.L.T. Kress (Kress, 2023) were all applied this designing principle.

In this paper, the research literature about intelligent weeding was reviewed from the aspects of obtaining the positions of seedlings and weeds and WEP. Their working efficiency, weeding rate, seedling damage rate and limitations were also reviewed. The purpose of this research is to merged cutting-edge detection technologies for crop and weed, as well as the WEP applied for all kinds of specific scenarios, it can provide the academic and design reference for the researchers in the fields of intelligent mechanical weeding, and it can also act as a quick start of the new beginners.

## 2 Literature and analysis

### 2.1 Search strategy

For the purpose of getting a comprehensive and objective assessment, the review examines the relevant literature as much as available. Initially, the popular literature index database was utilized for pertinent English-language works in this project, such as Google Scholar (https://scholar.google.com/), Scopus (http://www.scopus.com), and Web of Science (http://www.webofknowledge.com). The searching approach is as follows: Topic = “mechanical* weed control* intelligent” AND (Keywords added: intra-row weed control/robotic weeding/weeding robot). The earliest literature of the preliminary search was published in 1989, so the timespan = “1999-2022”, document types = “article+review article+meeting+dissertation thesis”, and 175 documents were obtained. Subsequently, Chinese literature was also been searched in the largest Chinese knowledge resource database in the world, CNKI (https://www.cnki.net). Topic = “mechanical* weed control* intelligent”. The earliest literature of the preliminary search was published in 2005, so the timespan = “2005-2022”, document types = “article+dissertation thesis”, and here are only articles from the EI and CSSCI are filtered in this article, and 121 documents were obtained. And then, these works were manually inspected to determine whether can be included or rejected. Specifically, the application scenarios had strict requirements, the relevant researches aiming at seedling and weed detection, as well as the specific weeding executive parts that served for crop farmland were included. The researches serving for other application scenarios were rejected, such as, the researches about lawn weeding, orchard weeding were rejected. Finally, we had collected 244 articles in total.

### 2.2 Research status analysis

#### 2.2.1 Annual published volume

**Figure 1:**
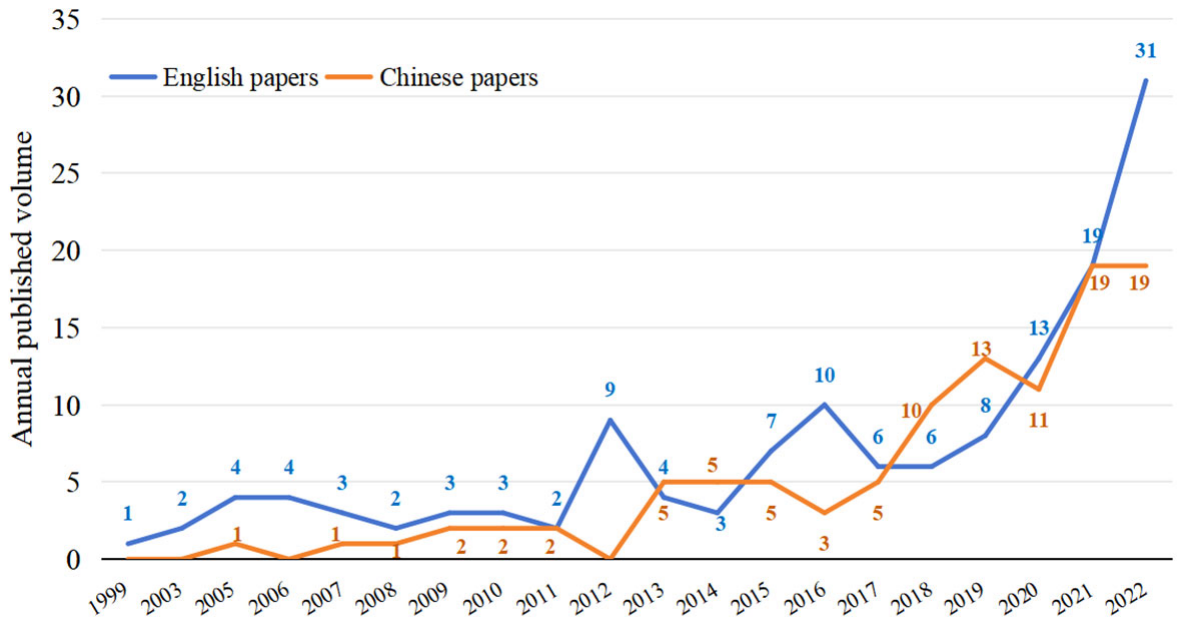
Number of published articles related to intelligent mechanical weeding. Among them, the number of articles from 1999 to 2003 and 2004 was 0.

The annual number of related research on intelligent mechanical weeding as shown in **Figure 1**. The number of documents shows an overall upward trend, fluctuating slightly in the middle. During the ten years from 2012 to 2022, the increase of the English papers was about350%, and the increase of Chinese papers was about 500%. After 2017, the number of publications has increased significantly, indicating that the research on intelligent mechanical weeding has gradually become a research hotspot.

#### 2.2.2 Research institutions and the authors

**Figure 2:**
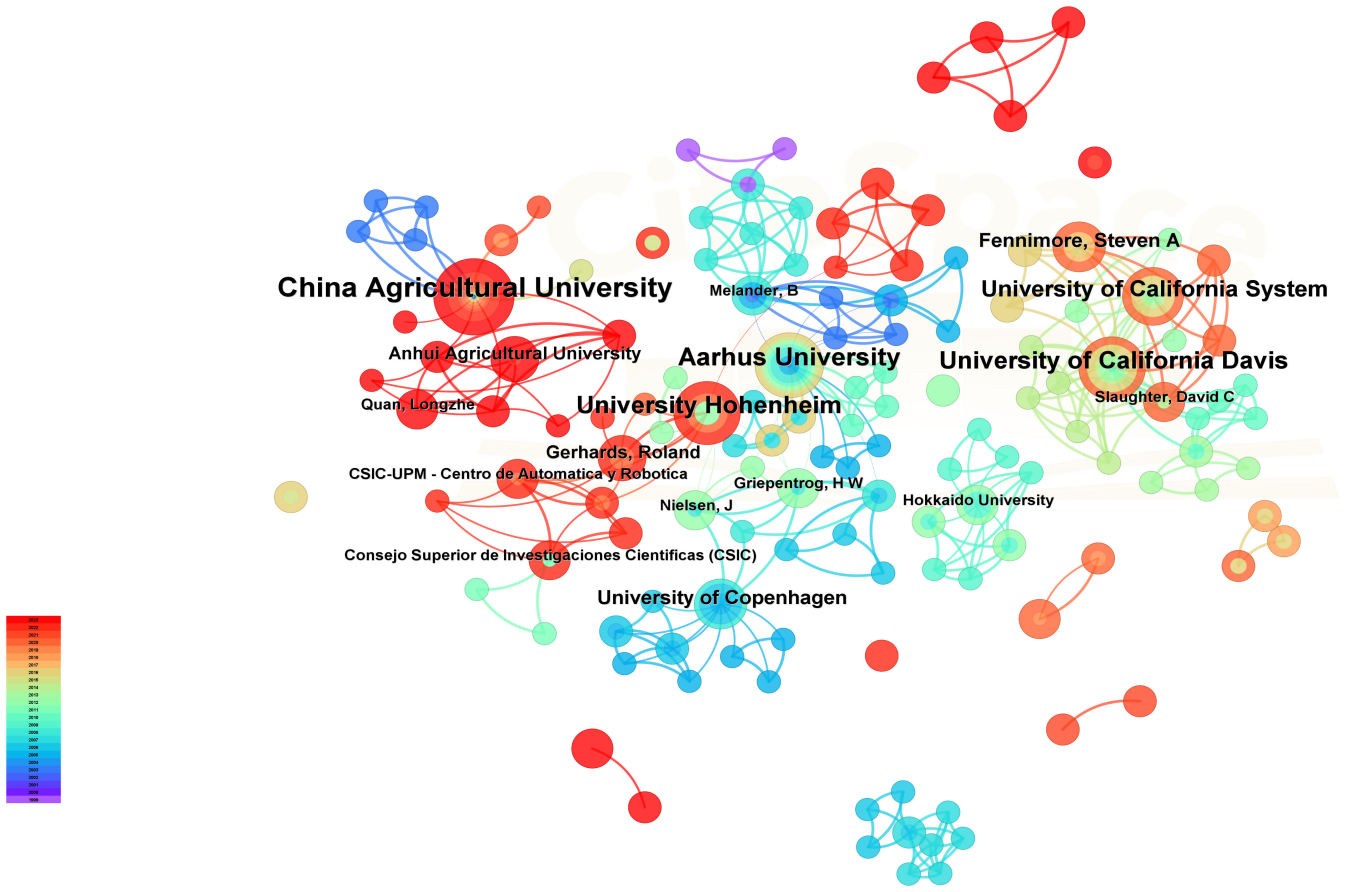
Institution and authors co-present knowledge graph. The size of the node indicates the number of publications, and the connection between the nodes indicates the connection between the institutions or the authors.

According to the statistics analysis of the national occurrence, the top five countries that the most frequently appear are China, the USA, Germany, Denmark and Japan. All of these countries are with good agricultural mechanization development and high degree of industrial automation. The relevant volume reached 40, 27, 23, 17 and 13 posts respectively. The organizations and authors of intelligent mechanical weeding are mainly concentrated in universities and research institutes, especially universities (**Table 1**), among which University Hohenheim, China Agricultural University, Aarhus University and others are in the forefront. Institution and the author map is as shown in **Figure 2**, it is visible that the research topic node size is small but the connection strength and the network density is higher, indicating that although the relevant research belongs to an emerging topic, the volume of submissions is not much, but the interaction between the research institutions or authors is closer, there is more academic cooperation between each other, and the research perspective is more concentrated.

**Table 1:** Top 10 research institutions and authors in the number of publications.

	Institutions		Authors	
	Name	Post volume	Name	Post volume
1	University Hohenheim	12	Gerhards, Roland	5
2	China Agricultural University	12	Fennimore, Steven A	5
3	Aarhus University	9	Slaughter, David C	4
4	University of California System	9	Norremark, M	3
5	University of California Davis	9	Griepentrog, H W	3
6	University of Copenhagen	6	Perez-ruiz, M	3
7	Consejo Superior de Investigaciones Cientificas	5	Czymmek, Vitali	3
8	Anhui Agricultural University	4	Nielsen, J	3
9	University of Bonn	4	Quan, Longzhe	3
10	University of Sevilla	4	Melander, B	3

## 3 Crop detection

The unique performance of intelligent mechanical weeding equipment is the ability to distinguish weeds and crops precisely. Mechanical weeding can be divided into two modes: one mode is identifying the crop, and another is identifying the weeds. As for the former mode, the WEP continuously performs the weeding operation and only performs the seedling avoidance operation if a crop is identified. As for the latter mode, the WEP performs removal action when weeds are detected. According to the technologies of distinguishing weeds and crops, there are three major subcategories, which are real-time detection technologies based on proximity sensors, geographical information system (GIS) and machine vision, respectively.

### 3.1 Detection technology based on proximity sensors

Proximity sensors are reliable and affordable. There are two types of proximity sensors which are physical contact sensors and non-contact sensors. As for physical contact sensors, they use a certain part to detect the crop position with the promise of no harm to crops [Jia et al. (2018)]. For non-contact sensors, they can detect crop positions without direct mechanical contact. Some examples of non-contact sensors include Hall proximity switches, ultrasonic sensors, laser sensors and X-ray sensors. One device mounted a Hall proximity switch on a single depth-limiting wheel [Zhou et al. (2018)]. The single depth-limiting wheel and the Hall proximity switch can measure the distance between the seedling and the depth-limiting wheel, and this information is sent to a micro-controller, which calculates the relative position between the weeding device and the target crop, and then a command is sent to a servo motor. The servo motor drives the weeding knife to rotate around the main rotating shaft, thus keeping the two weeding knives separate to avoid seedlings.

Another type of non-contact proximity switch is the ultrasonic sensor. Saber et al. (2015) developed a rotating pressure roller weeding device, which used two spinning pressure rollers to uproot weeds. The device has two ultrasonic sensors at the front and rear to detect crop positions. When the front ultrasonic sensor identifies an approaching crop plant, the weeding mechanism is quickly raised to pass the plant, and once it passes the crop plant, the rear ultrasonic sensor lowers the weeding part to the ground to work continuously. Cordill and Grift (2011) used a laser sensor to detect maize stalks. The laser sensor has a transmitter that emits four 3.5 mm-diameter visible red laser beams and a receiver that picks them up. By analyzing the pulse signals, the maize stalks can be distinguished from other objects, thus the exact location of the maize plant can be determined.

Haff et al. (2011) developed a tomato seedling detection technology by X-rays, which projected an X-ray beam perpendicular to the crop row and parallel to the soil surface through a portable X-ray source. The main stem of the tomato absorbs the energy of the X-rays, reducing the output voltage (signal), thus the tomato’s main stem can be detected even when it is obscured by weeds or crop foliage. The detection signal is then used for controlling a pair of intra-row mechanical weeding knives.

Proximity sensors are cost-effective, a mechanical travel switch is merely about ¥10 (e.g., https://item.taobao.com, accessed on 25 Jan 2024; https://www.tmall.com, accessed on 25 Jan 2024), and such configuration is really simple. They can be equipped in any necessary position according to specific requirements. But proximity sensors also have many limitations. Such as they can not distinguish crops and weeds, they can only tell that obstacles exist (Dou et al., 2024). If the weeds are higher than the mounting position, the non-contact sensor will emit an error signal. If the weeds also have strong enough stiffness, the contact sensor would also let the WEP avoid them rather than consider them as weeds.

### 3.2 Seedling pre-position based on GIS

Geographical Information System (GIS) is a comprehensive discipline that covers the content and knowledge of geography, cartography, mapping, management, remote sensing and computer science (Zhang and Cao, 2019). Its specific application in agriculture is depicted as follows, a GPS-equipped seeding machine will record the location information of each seed when sowing and draw a Geo-referenced seed map. The map can be used for determining the seedling location during the later field management. For instance, Norremark et al. (2008, 2012)developed an intra-row mechanical weeding system, which based on the RTK-GPS positioning technology to pre-locate the seed locations while sowing, then a geo-referenced seed map was created. The end-effector of the weeding machine will carry out seedling avoidance and weeding actions in accordance with the geo-referenced seed map during a subsequent weeding operation. Similar investigations could also be found in the studies of Perez-Ruiz et al. (2012).

Since GIS-based crop positioning detection is not impacted by outside factors, such as weather and light, it offers a high level of positioning accuracy. However, the technology is costly, and its requirements from supporting machines are higher (Zhu et al., 2009). Additionally, because the establishment of the map and the weeding operation do not occur at the same time, this technology is not applicable on-site, and new issues like crop loss and crop displacement that happened at the weeding operation site cannot be resolved quickly.

### 3.3 Machine vision technology

With the continuous rise of artificial intelligence technology, machine vision technology has been widely used to identify and locate crops and weeds. Machine vision technology can collect dynamic images of crops and weeds in real-time, and obtain the location information of seedlings and weeds at any moment (Sonka et al., 2002). Machine vision technology can be divided into two major divisions, which are image processing and deep learning. However, image-processing-centered technologies usually need to extract features such as color, morphology, texture and spatial distribution of crops and weeds in the form of vectors. Then, they select appropriate classifiers for detection and recognition, which are easily affected by factors such as light, shadow and mechanical vibration, making it difficult to meet the recognition needs of smart weeding operations (Chen et al., 2015). With regards to irregular-shaped object detection, image processing has a limited applicable ability, thus some other technologies should be introduced to expand the applicable ability. For example, post-processing algorithm. Zheng et al. (2017) developed a post-processing algorithm used for distinguishing maize seedlings from weeds after image preprocessing. Color indices were used to develop a classification model, and the nine optimal features were selected by principal component analysis to reduce the effect of illumination. Finally, support vector data description was used as a classifier to differentiate the maize from the mixes of different weed species. Results showed that the average accuracy was 92.14%.

**Figure 3:**
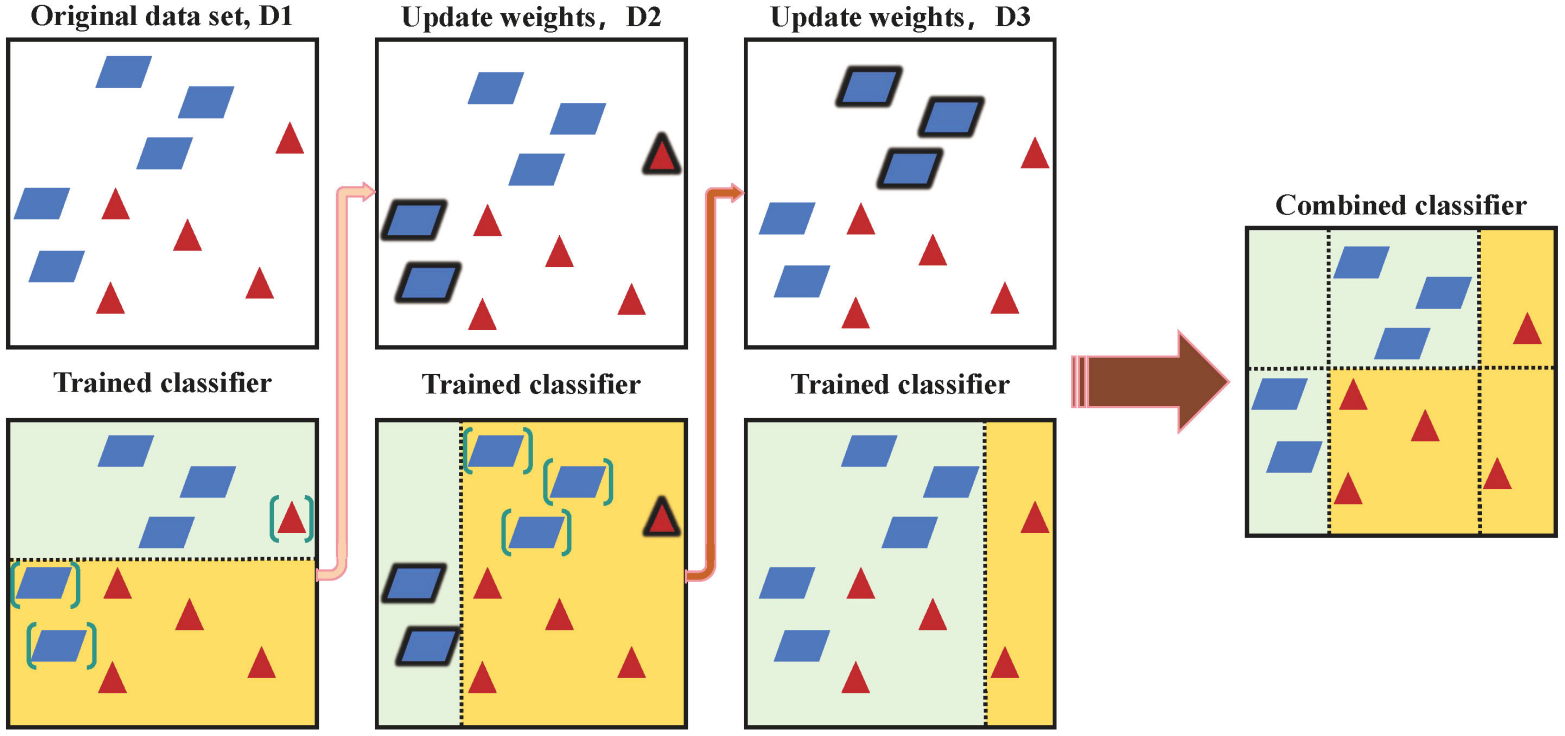
Schematic diagram of AdaBoost algorithm. Where D*i* denotes the weight distribution of the training data before the start of the *i*-th iteration, the trained classifier at the bottom is the weak classifier created in each iteration, the combined classifier is a strong classifier formed by combining all weak classifiers.

In order to compensate the flaw of merely utilizing image processing, and based on shallow machine learning methods, deep learning algorithms are proposed. Deep learning contains multiple hidden layers of multilayer perceptrons, and more abstract higher-level features are formed by combining lower-level features to express attribute categories or features to discover the distributed characteristics of the data (Marsland, 2015). Numerous deep learning algorithms have been widely used in crop and weed recognition studies, such as Artificial Neural Network (ANN), Support Vector Machine (SVM), Naive Bayes model, Ada Boost algorithm, Decision Tree, K-means, and so on (Sunil et al., 2022). ANN has a working mechanism similar to the human brain (McCulloch and Pitts, 1990), and SVM is a binary classification model (Cortes and Vapnik, 1995). The combination of these two technologies enabled the machine to distinguish weeds from sugar beets. Results showed the overall classification accuracy of ANN was 92.92%, where 92.50% of weeds were correctly classified (Bakhshipour and Jafari, 2018). Higher precision was obtained when the SVM was used as the classifier with an overall accuracy of 95.00% and 93.33% of weeds were correctly classified. In terms of sugar beet plant classification, the classification accuracies of ANN and SVM were 93.33% and 96.67%, respectively (Bakhshipour and Jafari, 2018). The AdaBoost algorithm has a nice performance in crop and weed classification (Freund and Schapire, 1997). Through multiple iterations and finally convergence, this algorithm can generate a classifier for seedlings and weeds. As shown in **Figure 3**, the AdaBoost algorithm adjusts the sample weights through each iteration, based on previous classification results, increasing the weight of incorrect classification and reducing the weight of correct classification, which ensures the overall classification accuracy in the end. Xu et al. (2020) investigated a natural weed identification method based on RGB image feature and depth feature fusion in wheat fields, the method extracted the color, location, texture and depth features from the RGB and depth images. The AdaBoost algorithm was used to synthesize and learn multiple classifiers. According to the experimental results, weeds were recognized with an accuracy of 88% during the tillering stage, and the recognition accuracy was 81.08% during the jointing stage.

Convolutional Neural Network (CNN) is one of the representative deep learning algorithms. The network structure of LeNet-5 which was proposed by Lecun et al. (1998) was one of the earliest CNN and the best version of the LeNet series. There are three convolutional layers in the model, which can reduce the parameter number of the whole network and learn the local feature information of the image. As well as two subsampling layers, which can reduce the resolution, make the features more abstract, and make the network converge more easily. As for reducing the resolution, it can make the computational speed faster and insensitive to offset and deformation. The implementation of LeNet established the structure of CNN, which is now the basis for a great number of neural networks (Patterson and Gibson, 2018). CNN has also been used to investigate the identification of crops and weeds (Ferreira et al., 2017; Teimouri et al., 2018; Camargo et al., 2021). CNN usually has a better performance combined with classifiers. For example, K-means is one of the representative classifiers, it can calculate the distance between each object and each seed cluster center, and assign each object to the nearest cluster center (Macqueen, 1967). Tang et al. (2017) constructed a weed identification model based on K-means feature learning combined with CNN to identify soybean seedlings and their associated weeds. According to the experimental findings, this approach obtained an accuracy of 92.89%, outperforming merely CNN with random initialization by 1.82%.

Crop detection and semantic segmentation are two main functions of deep learning algorithms when performing crop and weed recognition tasks. Crop detection can be considered as a combination of two tasks, target localization and classification, i.e., locating the position of an object in an image and identifying the class to which the object belongs (Viola, 2001). YOLO is one of the faster object detection models. In the YOLO algorithm, the core idea is to predict the presence of target objects in each grid, assign the corresponding class and confidence level by dividing the image several times, and finally combine the predictive results in all grids to select the final detection result, which is fast in execution and can achieve highly efficient detection. In order to address the stability problem of traditional image processing methods in the field complex background environment, the YOLOv5m model which incorporates a channel attention mechanism (SENet) was constructed by Li et al. (2022a). The mAP of the SE-YOLOv5m model on the test set was 90.66% (IoU 0.5), indicating the effectiveness of the SE-YOLOv5m model for detecting maize plants. The proposed SE-YOLOv5m model was able to infer at 20.4 ms on a GPU on an image with the size of 960 pixels × 540 pixels, which have the potential to be applied to embedded terminals. Evaluation under different weed proportions shows that different weed proportions in the field have no significant influence on the detection accuracy of the maize plant detection models. Of course, besides YOLO, some other deep learning models are also widely used in crop seedling identification tasks, such as Single Shot MultiBox Detector (SSD), Regions with CNN features (R-CNN), Faster R-CNN, and so on. Peng et al. (2019) found that the Faster R-CNN network by introducing a feature pyramid network in the Region Proposal Network (RPN) got better results compared with YOLOv3. Firstly, the model extracted image features by using a residual convolutional network, and then introduced a feature pyramidal network in the RPN to generate the target candidate frame, and finally realized the effective identification for cotton field weeds in complex backgrounds.

Semantic segmentation understands images at pixel level. This method identifies and classifies each pixel based on the semantic information contained in the image. Semantic segmentation can identify object locations and boundaries in images more accurately than Crop detection (Zou et al., 2021; Kamath et al., 2022; Wang et al., 2022; Ma et al., 2023). Fully Convolutional Networks (FCN) was the first semantic segmentation model, which was invented by Long et al. (2015), it segmented images by end-to-end training of CNN. Lottes et al. (2018) achieved semantic segmentation of sugar beet and weeds by using FCN with sequence information. The system relies on FCN with an encoder-decoder structure and incorporates spatial information by considering image sequences. Exploiting the crop arrangement information that is observable from the image sequences enables the system to robustly estimate pixel-wise labeling into crop and weed. Ma et al. (2019) proposed a SegNet semantic segmentation model based on FCN, which used VGG16 with fully-connected layers removed as the encoding part to achieve feature extraction. The encoding part consists of five stages with 13 convolutional layers, each stage contains a convolutional layer and a pooling layer, and the feature map size is halved after each stage. The decoding part is symmetric to the encoding part and also contains five stages with 13 convolutional layers, each stage contains up-sampling layers and convolutional layers, and the feature map size is doubled after each stage. All convolutional layers of the model are followed by Batch Normalization (BN) layers and ReLU activation functions, which allow the network to converge faster and improve the nonlinear representation of the network. The experiments showed that the method achieved high classification accuracy in the segmentation of rice seedlings and weeds, with an average accuracy of 92.7%.

Overall, machine vision technology has become the mainstream technology compared with proximity sensors and GIS based pre-positioning technologies, it can accurately locate seedlings and grass through techniques such as image processing and deep learning, and laying a technical foundation for subsequent weeding operations. However, machine vision technology requires high technical support and maintenance, and factors such as lighting, soil moisture, and background in farmland may affect the recognition effect of machine vision technology. Therefore, it needs to be adapted and adjusted in different environments. Machine vision technology requires high research and equipment costs, which may be difficult for some small farmers to afford. This will be further discussed in depth in the following text.

## 4 Weeding executive parts

There is a metaphor, the detection technology for seedlings and weeds is equivalent to the human eyes, the control system is equivalent to the human brain, and the weeding executive parts (WEP) are equivalent to the human hands. The weeding scenarios can be divided into paddy fields and dry fields, so the WEP are designed to meet their specific soil conditions.

### 4.1 Paddy fields WEP

Compared with dry fields, which are relatively fertile and compacted, paddy fields have the characteristics of high moisture content, low strength, and high viscosity due to long-term immersion. In order to avoid weed tangling and soil adhesion, the WEP for paddy fields are usually designed into a spring-tooth shape. Tian (2022) demonstrates a paddy cage weeding device, the intra-row WEP is a spring-tooth shape, and the weeding parts will comb the paddy seedlings and weeds indiscriminately. Since the paddy is stronger than the weeds during the tillering period, paddy roots are deeper than the common weeds (Hance and Holly, 1991), so the weeds can be removed and paddy seedlings will be retained. However, although the damage to crops is relatively small, it still has harm to crops. In order to realize non-contact intra-row weeding, Tao et al. (2015) designed an intra-row WEP, which removes the intra-row weeds by WEP rotating between two seedlings, the rotational movement cuts the weed roots below the soil surface, as well as stirs and flips the mud. The seedling avoidance function of the intra-row WEP is realized by manual adjustment, operators should control the intra-row WEP to forward along with the row, and pay special attention to letting the WEP enter and leave the intra-row spaces, so as to avoid the contact between the WEP and seedlings.

### 4.2 Dry fields WEP

Dry fields have the characteristic of less soil adhesion compared with paddy fields, so not only spring-tooth WEP, but also large-contact-area WEP can be applied in dry fields. In terms of inter-row weeding, hoes are widely applied, and fingers are also widely applied in intra-row weeding. Besides the non-powered WEP above, some powered intra-row WEP are also hotspots, such as brushes, swing executive parts and rotational executive parts.

**Figure 4:**
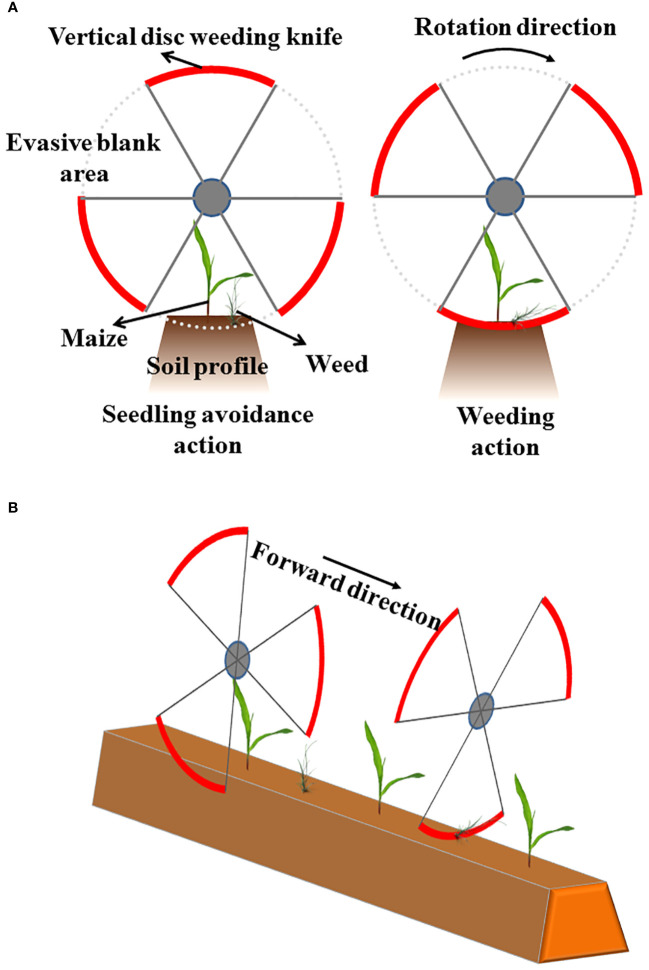
Structure schematic diagram of disc weeding knife with vertical rotation. **(A)** Front view. **(B)** Isometric view.

- Hoe WEP. The weeding mechanism of hoes is as follows, while they are dragged by power machines and stretched into the soil, the inter-row weeds will be removed along with their proceeding. Generally speaking, weeding hoes have two types, the sweep and the ducksfoot (Pullen, 1995; Godwin et al., 2007). Usually, the hoe unit has its own depth limit wheel and is attached to the main frame by a spring-loaded parallel linkage, thus ensuring operation at an accurate depth. The blade of sweep hoes is either an ‘L’ or ‘A’ shape and removes weeds by cutting their roots just below the ground at a depth of about 25 mm (Pullen and Cowell, 1997). The ducksfoot blade differs from the sweep because of its raised profile, the raised profile has the function of burying and mixing the weeds. The ducksfoot blade is usually attached to a spring tine. Han (2011) designed a horizontal disc weeding machine with a ducksfoot actuator. It is driven by the ground wheel and passively loosens the soil, breaks the soil consolidation in the seedling row and removes weeds. It has a monomeric profiling function and can get different cultivation depths by adjusting the installation height of the fixed shovel handle.
- Spring-tooth WEP. The spring-tooth WEP can be added to an inter-row cultivator to increase the function of intra-row weeding. One kind of the spring-tooth WEP is composited by spring-loaded steel rods on each side of the row. The executive parts can undercut small weeds between two maize seedlings. Since there exists stiffness difference between maize seedlings and weeds, the spring tines will separate when they encounter the maize seedlings, rather than damage them. Han (2011) invented a combined spring-tooth intra-row weeding machine with six groups of spring teeth divided into two sections at the front and rear. Each group of spring teeth stretches vertically to a depth of 20 to 40 cm into the ground due to gravity.
- Finger-shaped WEP. The finger-shaped WEP is usually composed of two discs with opposite directions, on which finger-shaped steel teeth or finger-shaped rubbers are installed (Godwin et al., 2007). While working, the disc rotates under the soil friction force, causing the finger-shaped structure to enter the soil and damage the weed roots. Owing to the space between the two opposite discs that can accommodate the diameter of crop seedlings, there will not be seedling damage if the forward direction is guided correctly.
- Brush WEP. The brush WEP are composed of flexible polypropylene brush discs that rotate powered or non-powered at a depth of 20-30 mm into the soil surface (Kouwenhoven, 1997; Godwin et al., 2007). The effect of the brush WEP is to lift the weeds out of the soil, breaking the stems and exposing the roots, leaving them vulnerable to drying out. Brush discs can be easily assembled into units of the desired width and spacing for different crops. Higher rotational speed means better weeding performance, while resulting in dust in dry conditions at the same time.
- Swing WEP. Swing WEP are designed to achieve seedling avoidance and weeding by driving the executive parts in a reciprocating motion, which can be divided into single and double swing types depending on the components. The automatic hoeing machine developed by Drimac (2023) used a single-swing design, which removed weeds from the intra-row by driving the weeding knife in a reciprocating motion. WEP with double swing design are more common, such as Home (2003); Perez-Ruiz et al. (2012); Perez-Ruiz et al. (2014), Robovator intra-row WEP (Midtiby et al., 2012; Melander et al., 2015; Poulsen, 2023a), ICWeeder interplant WEP (Steketee, 2023), and Zhou et al. (2018) all used this design principle. The intra-row weeding knives have two working states. The inside tips of the blades touched each other, which was defined as the ‘closed’ state. With the knives in the closed state, all weeds in the central area of the crop row were killed. The other state was defined as the ‘open’ state. By actuating the related switches (e.g. pneumatic valve, solenoid valve, motor, etc.), each linkage arm and its associated intra-row knife blade were positioned away from the seedling, creating a knife-free, uncultivated area centered around the seedling. With the intra-row knives in the open state, all plants growing within the central region were not killed, while all plants growing outside the central region were killed.
- Rotational WEP. Rotational WEP achieve seedling avoidance and weeding by driving the WEP to rotate periodically according to a certain pattern, which can be classified into horizontal rotation and vertical rotation. Quan et al. (2022) designed a disc weeding knife with vertical rotation based on a wheeled mobile platform, where each disc consisted of three weeding knives and the gap between the weeding knives served as a space for crop avoidance (**Figure 4**). A similar design could be found in the publication of Gobor (2013). Intra-row WEP with a vertical rotation design is more common, such as disc-hoe WEP (Garford, 2023), and the cycloid hoe WEP (Norremark et al., 2008, 2012), etc. Garford (2023) designed a disc-hoe with an arc-shaped notch, this kind of WEP realized weeding by rotating the notched disc knife, and the seedling avoidance function is realized by a control program, which lets the notch towards the seedling all the time when a crop is detected. Since the notched disc knife is stretched into the soil all the time, along with the forward of the power machine, it achieves weeding around the crop. The cycloid hoe has some tines, the tines will stretch into the soil, and the root-soil complex of weeds will be brought out. The cycloid hoe is suitable for inter-row weeding, and it can also be applied in an intra-row weeding machine if combined with a seedling-avoidance control strategy, which allows the seedlings to pass through the tine gaps.

Through the above literature, it could be concluded that each WEP was invented to meet some specific weeding scenarios, the summary is shown in **Table 2**.

**Table 2:** Weeding executive parts and their characteristics.

Types	Advantages	Disadvantages	Applications
Hoes (Pullen, 1995; Godwin et al., 2007)	More effective and high operating speed	The risk of crop burial	Inter-row weeding for dry fields and paddy fields
Spring-tooth (Han, 2011)	Simple structure and easy to cooperate with cultivators	Accurate row-controlling requirements	Intra-row weeding for dry fields and paddy fields
Finger (Godwin et al., 2007)	Simple structure, light weight and good intra-row weeding performance	Accurate row-controlling requirements	Intra-row weeding for dry fields
Brush (Kouwenhoven, 1997)	Unique ability of working in high moisture soil	Power required Relative high seedling damage rate Dust in dry conditions	Omnibearing weeding before seedling; Inter-row weeding for dry fields
Swing type (Drimac, 2023)	Simple to design and manufacture High operational reliability	Power required Greatly influenced by the forward speed and the planting space Most of them need the assistance of detection control systems	Intra-row weeding for dry fields
Rotational type (Garford, 2023)	Unique ability of working in nearly all kinds of soil conditions	Power required Most of them need the assistance of detection control systems	Intra-row weeding for dry fields

## 5 Discussion

### 5.1 Comparison of various weed control methods

In terms of working efficiency, intelligent weeding machinery has significant advantages over manual weeding. First, it can work continuously for a rather long time while ensuring the operational quality. For example, the weeding robots created by the German company K.U.L.T. Kress has an operating width of 1.5 m to 3.5 m, an operating speed of up to 5 km·h^-1^ and an operating capacity of up to 1.5 ha·h^-1^ (Kress, 2023). AgBot II and Dahlia Robotics can realize uninterrupted autonomous navigation and weeding by carrying solar-power systems, operate an average of about 2.7 ha per day, and their efficiency can be 5-8 times that of personal manual operation (Garford, 2023; Kress, 2023; Robotics, 2023; Poulsen, 2023b). As for traction-type equipment, it can carry several weeding units, so it can realize the simultaneous weeding operation of multiple rows, and its efficiency can be further improved. Completing the heavy and monotonous weeding work with intelligent weeding machinery is not only a more effective method, but also serves to optimize human resources and reduce labor costs. Applying intelligent weeding machinery is important for promoting agricultural modernization and technological progress.

Although intelligent weeding machinery can work continuously with excellent quality for a long time, it is still impossible to perform weeding operations at relative high speeds (average operating speed is less than 3 km·h^-1^), and the production efficiency is far below chemical weeding (average operation speed exceeding 12 km·h^-1^). The crop or weed detection speed is a major factor that limiting the weeding operating speed. For example, when applying a non-contact proximity sensor for crop recognition and localization (as described in Section 2.2), since proximity sensors have a relative close detection range, the actuators have limited error room in executing movements, as well as these proximity sensors have a relative slower reaction frequency, so they can properly work only when the weeding machine has a relative slower forward speed, ensuring the WEP have sufficient response time to execute the movement (Ye et al., 2020). For example, Cordill and Grift (2011) used a laser sensor to detect maize seedlings, limited by hardware and the algorithm, the weeding machine’s highest speed was only 0.1 m·s^-1^. In terms of detecting seedlings based on machine vision technology (as described in Section 2.3), since the detection accuracy is negatively correlated with the forward speed, it is necessary to ensure higher-quality detecting results by reducing the forward speed. In terms of the weeding machine designed by Chang et al. (2021), its best operation speed was only 0.15 m·s^-1^, and when the machine’s speed exceeded 0.2 m·s^-1^, the operational quality decreased sharply, which was reflected by the increased crop damage rate and reduced weeding rate. Generally speaking, in order to achieve a higher weeding quality, it has to sacrifice weeding efficiency.

WEP’s execution speed is another factor that affects the weeding efficiency. As described in Section 3, different WEP (blades, brushes, rollers, etc.) remove weeds in different ways (rotation, vibration, or rolling), and the different actuation speeds determine weeding efficiency directly. The longer the execution cycle, the slower weeding efficiency.

In addition, mechanical weeding is not as effective as chemical weeding currently. Barbas et al. (2020) conducted an experiment to compare the weeding effectiveness between mechanical and herbicide treatments on weed densities and biomass. The experimental results showed that chemical methods were more effective than mechanical methods in reducing weed densities and biomass. Cao et al. (2023) reached similar conclusions, they found that owing to the fact that mechanical weeding may not completely remove the weed root system, it does not address the underlying issues that lead to weed revival. Therefore, there requires additional solutions in conjunction with mechanical weeding, such as repeated applications of mechanical weeding or using a combination of mechanical and biochemical methods, in order to manage weed populations more effectively over a relative long term.

### 5.2 Influential factors of weeding rate and crop damage rate

Stable weeding rate and crop damage rate are key factors in the application of intelligent mechanical weeding technology. The stable weeding rate ensures a clean crop growing environment and avoids weeds competition with crops for resources such as water, nutrients and sunlight, which can improve the yield and quality of crops, and at the same time, the damage caused by machinery should be avoided. However, in current practical applications, there is often a certain correlation between the weeding rate and crop damage rate, and the operation effect of the technique is vulnerable to the impact of the operation scenario, soil conditions, cultivation agriculture, crop characteristics or weather factors. It is a challenge to achieve a stable stable weeding rate and crop damage rate.

Generally speaking, it is difficult for intelligent weeding equipment to operate in paddy fields compared with dry fields, so the performance in paddy fields is also suboptimal. Substantial intelligent mechanical weeding-related literature showed that the paddy weeding rate is generally around 80%, and the crop damage rate is around 4%. While the dry field weeding rate is rather higher, the weeding rate is generally between 90% to 95% and the crop damage rate is between 2% to 6% (Bakker et al., 2011; Perez-Ruiz et al., 2014; Quan et al., 2022). For example, in the study of Jia et al. (2018), a seedling avoidable weeding device for inter-tillage maize based on the proximity switch sensor showed an average weeding rate of 94.7%. Similarly, as for the study of Chang et al. (2021), the experimental results showed that when the forward speed was lower than 0.15 m·s^-1^, the weeding rate was 92.6%.

Beyond the operational scenarios, in actual field operations, the complex ground conditions are also important factors that affecting the weeding performance of intelligent weeding equipment, such as the complexity of the terrain, soil moisture and surface cleanliness. The more complex the terrain, the lower the weeding performance, because higher complex ground conditions let the weeding equipment steer, turn around and do other operations more frequently, which may decrease weeding rates and increase seedling damage rates. A related study showed that soil moisture has a greater impact on the operating effect (Rayhan et al., 2021). Mechanical weeding works best in dry soil conditions because weeds can be uprooted or cut off easily (Yan, 2021). And surface cleanliness has a profound influence on the weeding rate and seedling damage rate as well. Sun (2020) conducted a comparative experiment which showed that the relative lower residue-remained plot increased the weeding rate by 2.3% and 0.8% in terms of inter-row and intra-row weeding, respectively. Similarly, when the field is filled with too many large soil clods and rocks, etc., intelligent weeding equipment will lose its operational efficiency, and the equipment’s life will also be affected greatly (Hiesl and Benjamin, 2013).

The influence of environmental factors is also cannot be ignored. Such as wind direction and intensity, illumination intensity and so on. It always requires specific conditions for the best results (Jeon et al., 2011; Hu et al., 2013). For example, strong winds can cause crops sway, overlap or shade, further increasing the difficulty of positioning (Chen et al., 2020). And in the research of Parra et al. (2020) and Yu et al. (2019), they also hold the similar viewpoint that environmental factors have an impact on the accuracy of identification. Even lighting conditions can affect the appearance and visibility of the target object, leading to issues like bright spots or shadows in the image, which affect the detection of the target object (Jeon et al., 2011). Chang et al. (2021) and his colleagues also verified this conclusion through collected images under different lighting conditions. Therefore, in order to ensure the stability and reliability of machine vision systems, it is necessary to consider the lighting changes and take corresponding measures, such as using light compensation algorithms, adjusting the camera positions, regular calibration of the machine vision system, etc. Furthermore, in order to be applied in the field scenario, special equipment is required. For example, to deal with silt in paddy fields or dust in dry fields, the image recognition equipment needs to have high dust and waterproof grade.

**Figure 5:**
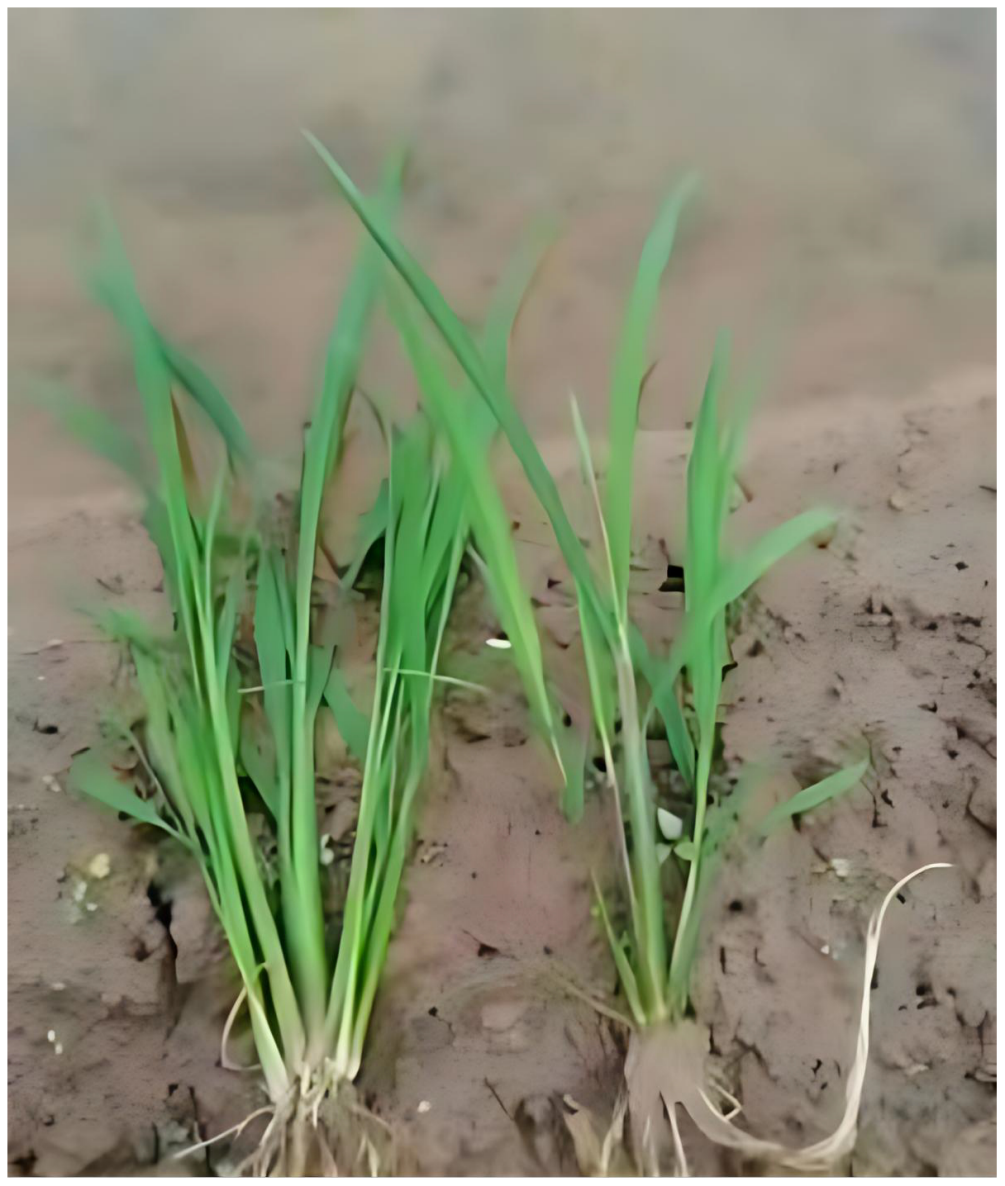
The gramineous weed *Echinochloa crus-galli* (L.) P. Beauv (left) and rice seedlings (right). They are highly similar in terms of appearance, which poses great difficulties for image recognition technology to distinguish them.

Furthermore, due to the intelligent weeding equipment typically relies on image recognition technology to identify weeds and distinguish them from crops (Chang et al., 2021). However, this technology may not be accurate enough to identify all types of weeds, particularly if there are some visual similar characteristics between the crops and the weed. For example, rice and gramineous weeds (**Figure 5**) all have long and narrow leaves, and inadequate morphological differences cause a hassle for current machine vision technology to distinguish them (Jiang, 2019). Even for the same crop species at different growth periods, the operation effect of intelligent weeding machinery is not the same. This is because the crop’s appearance will change all the time. Accordingly, the need for a full-cycle sample collection method to avoid the influence of crop appearance’s dynamic changing has been mentioned in numerous studies. These tests confirmed that the detection accuracy of crops and weeds is limited by the growth stages (Han, 2011; Jiang, 2019; Feng, 2020; Zhang, 2021).

In addition to the above non-machine influencing factors, the WEP can also affect the weeding and seedling injury rates. In Han’s study, two WEP were designed but the inter-row weeding performance was quite different (Han, 2011). Under optimal operating conditions, the horizontal disc inter-row weeding unit (hoe type) achieved weeding rate of 73.1% and crop damage rate of 4.41%, while the assembly multi-finger intra-row weeding mechanism were 87.6% and 2.73%, respectively.

But we are confident that with the development of science and technology and manufacturing, the performance of intelligent grinding equipment will become more and more stable, and the good stability and balance between grinding rate and seed rate will be achieved, so that intelligent mechanical grinding technology can really play its advantages and intelligent development of agriculture.

### 5.3 Limitations of the application

Mechanical weeding technologies offer several advantages over biochemical weeding methods, such as being environmentally friendly and reducing the risk of biochemical contamination. It is an advanced and promising agricultural technology that has the potential to revolutionize agricultural practices (Westwood et al., 2018). However, there are also several limitations that must be taken into consideration.

**Figure 6:**
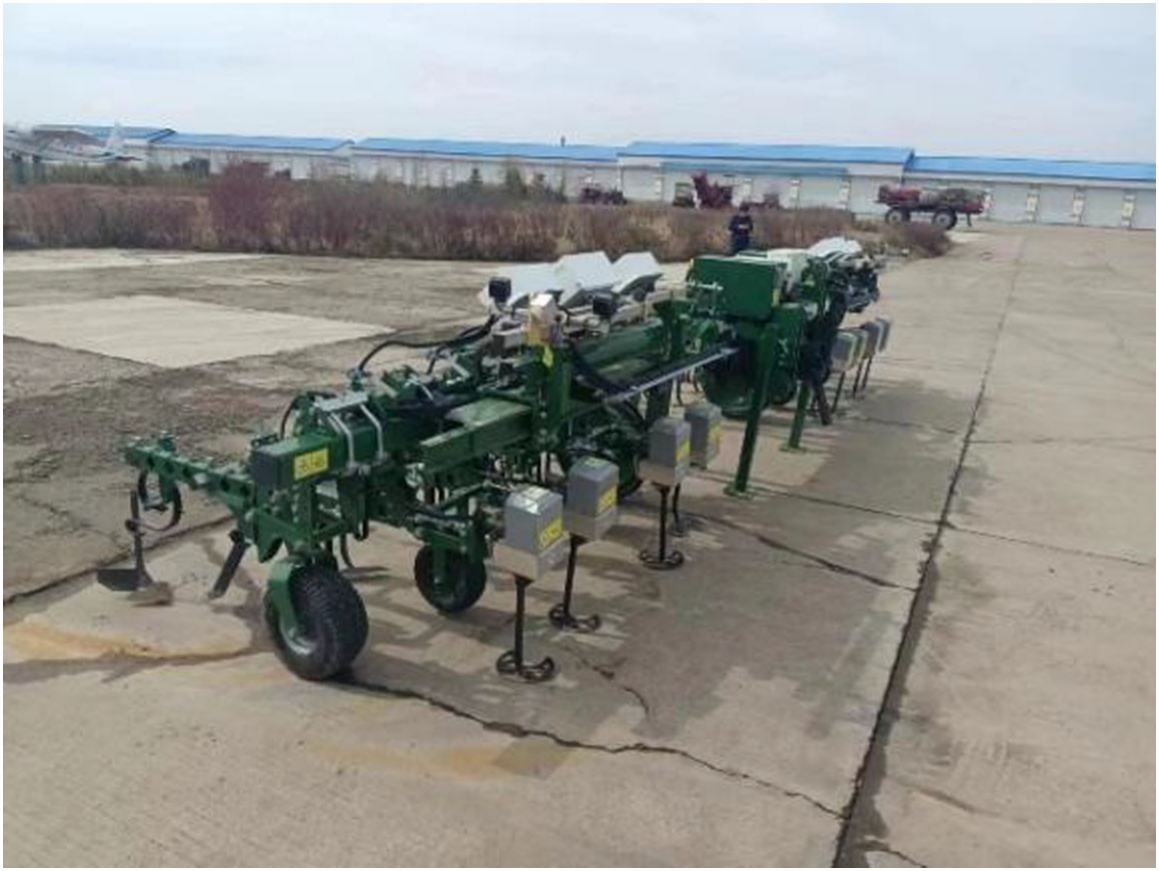
Intelligent mechanical weeding machine purchased by Red Star Farm in Heilongjiang Province, China.

1. High technology costs. The higher initial investment and ongoing maintenance costs directly limit its accessibility and promotion in markets for smaller growers or operations with limited resources (Cox et al., 1999). Intelligent mechanical weeding methods, such as robotic weeding, can be expensive to implement, requiring significant upfront funds in terms of technology research and infrastructure development. Moreover, the operations of intelligent weeding equipment require a wide range of skills, such as mechanical operation, electronic knowledge, crop field management and maintenance skills to ensure normal operation and operational quality. This means farms require more professional labors, particularly for larger farms (Gazoulis et al., 2021; Schlinker, 2021), which increases the labor costs. Besides, intelligent mechanical weeding equipment is the integration of multiple technologies, and due to the compatibility among different equipment, more capital investment is also needed to address the issue of technology compatibility (Alba et al., 2020; Machleb et al., 2021; Hosseini et al., 2022). During the research investigation, the authors learned that Red Star Farm in Beian City, Heilongjiang Province, China once spent ¥ 2.8 million to purchase an intelligent maize weeding machine based on image recognition in 2021 (As shown in **Figure 6**), and it also needs to be equipped with professional operators and maintenance technology, which further increases the application cost (Bawden et al., 2017). Consequently, this cost may be prohibitively high for small-scale farmers or those with limited financial resources, meaning that this technology may only be accessible to economical capable agribusinesses.
2. Employment reduction. The rapid development of intelligent weeding technology has enabled agricultural equipment to perform automatically and with high quality, but as intelligent weeding equipment becomes more widely used, it may have some impact on employment rates. First of all, traditional farmers or agricultural workers need to invest a lot of time and effort to perform weed removal, but the advent of intelligent weeding technology can replace the human resources (Van Der Weide et al., 2008; Kunz et al., 2018). Secondly, the application of intelligent weeding technology will improve the relevant skills, and the higher threshold will eliminate traditional agricultural practitioners who cannot meet the higher technical requirements, compressing the population eligible for the position.
3. Production impact. Theoretically, intelligent mechanical weeding can effectively control weed populations, promote normal crop growth and improve yields. However, in practice, the effect of intelligent mechanical weeding on crop yield is still a controversial topic compared with chemical weeding. Some relevant studies have shown that compared with chemical weeding, intelligent mechanical weeding could obviously increase the activities of superoxide dismutase and peroxidase in the uppermost leaves of rice plants, thus effectively increasing the crop yield. Liu et al. (2023) verified the above conclusions by three field experiments in 2020 and 2021. Three experimental fields were set up in South China, the results showed that mechanical weeding significantly increased the tiller numbers by 7-23% and grain yield by 2-11% at the harvest maturity stage compared with the chemical weeding. However, there also existed the viewpoint that crop yields tended to decrease as herbicide usage decreased (Fang et al., 2022). For example, in a study conducted by Kunz et al. (2018), they mentioned that compared with untreated control, there was a significant increase in crop yield with either chemical weeding or intelligent mechanical weeding treatment. However, when comparing between the two treatments, mechanical weeding yielded an average of 48% less than chemical weeding. Of course, there are also numerous researchers who consider that there is no significant difference between chemical and mechanical weeding, including Pannacci and Tei (2014); Fogliatto et al. (2019); Machleb et al. (2021); Jiao et al. (2022) and so on. They explored the effects of both treatments on sugar beet, maize, soybean, and snap bean crops, respectively, and obtained similar conclusions, indicating that the effects of mechanical weeding on yield and quality were essentially the same compared with conventional herbicides. So the impact of intelligent weeding on crop yield is unclear and requires comparative analysis based on specific situations and field measurements.

### 5.4 Future technology trends

Green and precise weed control is a crucial element in the advancement of green agriculture and precision agriculture. According to the existing literature, the applications of intelligent weeding machinery in field scenarios is shown in **Table 3**, they are mainly divided by crops such as vegetables, maize and soybeans. In addition, the table summarized the detection network model, weeding execution parts, operation efficiency and weeding effect evaluation in different crop-field application scenarios.

**Table 3:** Statistics on the application status of the intelligent weeding machinery.

References	Crop type	Detection method	WEP	Application/Performance
Jiang et al. (2023)	lettuce	optimized SPH-YOLOv5x model Accuracy = 95%	A dilution knife in the vegetable diluter	Efficiency = 3.28 km/h Weeding rate = 80.25%
Ye et al. (2023)	Soybean	laser ranging sensor Accuracy = 95%	A elastic Comb Reciprocating a Soybean Plant-to-Plant Seedling Avoidance and Weeding Device	Efficiency = 0.31 m/s Weeding rate = 98.2% Seedling injury rate = 1.69%
Visentin et al. (2023)	lettuce	Deep Neural Network (ResNet18) Accuracy = 98% (crop) Accuracy = 98% (weed)	A gripper	Weeding rate = 85% Seedling injury rate ≤5%
Quan et al. (2022)	Maize	YOLOv3 network Accuracy = 98.5% (maize) Accuracy = 90.9% (weed)	A blade weeding knife A wedge weeding knife A plough-surface weeding knife	Efficiency = 1.8km/h Weeding rate = 85.91% Seedling injury rate = 1.17%
Lottes et al. (2017)	Carrot, sugar beet	A system that performs vegetation detection, local as well as object-based feature extraction, random forest classification, and smoothing through a Markov random field to obtain an accurate estimate of crops and weeds.	A multi-propose field robot by BOSCH DeepField Robotics	Weeding rate = 93.86%
Astrand and Baerveldt (2002)	Sugar beet	gray-level vision system and color-based vision system Accuracy = 77% (Sugar beet) Accuracy = 87%(Weed) Guidance Accuracy = ± 2 cm	rotating wheel that is rotated perpendicular to the row line.	When the distance between the plants was about 17 cm, the robot was able to recognize all the sugar beet plants and the weeding tool worked well.

The statistics listed above indicate that intelligent machinery weeding technology is still in proceeding. In order to advance the real applications, we recommend that researchers need to invest more efforts in the following areas:

1. Increase the universality of machines. To adapt to different types of crops and soil conditions, it is necessary to design and develop intelligent mechanical weed control tools that are versatile. This can reduce the investment cost for farmers and crop growers, and improve the applicability of the technology in various agricultural environments.
2. Improve the accuracy of machine visual detection models. Intelligent mechanical weed control technology relies on machine vision detection models to distinguish weeds from crops. Continuous improvement of machine learning algorithms and training datasets is needed to enhance the accuracy and robustness of the detection models.
3. Increase the weeding efficiency. The operational efficiency of weed control equipment is a key factor in promoting the practical application of intelligent mechanical weed control technology. By improving the working speed, stability, and operability of weed control equipment, human resources and operating time can be effectively reduced, thereby enhancing the productivity of crops.
4. Increase the operational quality. Intelligent mechanical weed control technology should not only meet the quantity demands of crops, but also ensure the quality of operations. Researchers need to continue improving the weeding accuracy and operational precision of weed control equipment to ensure minimal damage to crops and maximize weed removal.
5. Reduce the technological costs. Currently, the cost of intelligent mechanical weed control technology is relatively high. In order to promote its widespread adoption, researchers need to make efforts to reduce the cost of technology, including research, production, and maintenance costs of machinery and related technologies. This will make it easier for farmers and crop growers to adopt intelligent mechanical weed control technology and benefit from it.

## 6 Conclusions

Intelligent mechanical weeding is a complex work, which needs the collaboration of Crop detection, weeding executive parts movements and so on. Nowadays, mechanical sowing is widely applied, which leads the crop positions to be uniform, so the Crop detection of aiming crops is easier than that of aiming weeds. In other words, precisely avoiding the crop seedlings and then executing weeding movements in the other areas is a better strategy for completing mechanical weeding.

With respect to the crop detection, although proximity sensors are cost-effective, they can not distinguish crops and weeds, GIS-based seedling pre-positioning technology can not figure out onsite issues such as crop loss or crop displacement. Thus, we deduced that the machine vision technology will be the mainstream in the future, and reducing environmental interference, improving robustness and detection accuracy will always be the right development directions. All kinds of mechanical WEP have their own advantages in their special working scenarios, although some crops could tolerate the indiscriminate weeding treatments during some certain growing stages, the WEP which have the seedling avoidable function would be more popular, owing to the special protection mechanism.

Although intelligent mechanical weeding has some limitations currently, nobody can deny that intelligent mechanical weeding has so many advantages over biochemical and hand weeding. It is inevitable that intelligent mechanical weeding will become a mainstream weeding method in the future, the relevant technologies and key components will have a flourishing development, it is only a matter of time. However, to achieve these developments, we proposed some suggestions as follows:

1. Research and development of equipment that suitable for various crops or scenarios.
2. Precise detection of crops in complex field environments.
3. Creation of high-performance intelligent weeding machines.
4. Research of corresponding weeding execution parts.
5. Promote key technological innovations and reduce technology costs.

We insist that when the technology costs will be lowered, the robust is improved in the near future, intelligent mechanical weeding equipment will be widely used.

## Author contributions

MX: Writing – original draft, Investigation, Visualization, Writing – review & editing. MQ: Writing – original draft, Investigation, Visualization, Writing – review & editing. GW: Writing – original draft, Funding acquisition, Investigation, Visualization, Writing – review & editing. ZM: Writing – original draft, Investigation. XC: Writing – review & editing, Supervision, Validation. ZZ: Writing – review & editing, Methodology. JQ: Writing – review & editing, Supervision. XG: Writing – review & editing, Project administration. HL: Writing – review & editing, Software. HJ: Writing – review & editing, Funding acquisition.
